# Significant Inter- and Intralaboratory Variation in Gleason Grading of Prostate Cancer: A Nationwide Study of 35,258 Patients in The Netherlands

**DOI:** 10.3390/cancers13215378

**Published:** 2021-10-27

**Authors:** Rachel N. Flach, Peter-Paul M. Willemse, Britt B. M. Suelmann, Ivette A. G. Deckers, Trudy N. Jonges, Carmen van Dooijeweert, Paul J. van Diest, Richard P. Meijer

**Affiliations:** 1 Department of Oncological Urology, Cancer Center, University Medical Centre Utrecht, 3508 Utrecht, The Netherlands; r.n.flach-2@umcutrecht.nl (R.N.F.); p.m.willemse-3@umcutrecht.nl (P.M.W.); rmeijer6@umcutrecht.nl (R.P.M.); 2 Department of Medical Oncology, Cancer Center, University Medical Centre Utrecht, 3508 Utrecht, The Netherlands; b.b.m.suelmann@umcutrecht.nl; 3 The Nationwide Network and Registry of Histo- and Cytopathology in The Netherlands (Foundation PALGA), 3991 Houten, The Netherlands; ivette.deckers@palga.nl; 4 Department of Pathology, University Medical Centre Utrecht, 3508 Utrecht, The Netherlands; g.n.jonges@umcutrecht.nl (T.N.J.); c.vandooijeweert@umcutrecht.nl (C.v.D.)

**Keywords:** Gleason grading, pathology, prostate cancer, interlaboratory variation

## Abstract

**Simple Summary:**

Gleason grading of prostate cancer is essential for treatment strategies and patient prognosis. Previous studies showed grading variation between pathologists when grading prostate cancer. Our study analyzed the presence and extent of grading variation between and within pathology laboratories in The Netherlands. In our nationwide retrospective study, we analyzed prostate needle biopsy reports of 35,258 patients in The Netherlands graded by 40 pathology laboratories. We found a considerable variation between and within pathology laboratories, as over half of the laboratories graded significantly different from the national mean. This likely affects treatment strategy and prognosis assessment of prostate cancer patients.

**Abstract:**

Purpose: Our aim was to analyze grading variation between pathology laboratories and between pathologists within individual laboratories using nationwide real-life data. Methods: We retrieved synoptic (*n* = 13,397) and narrative (*n* = 29,377) needle biopsy reports from the Dutch Pathology Registry and prostate-specific antigen values from The Netherlands Cancer Registration for prostate cancer patients diagnosed between January 2017 and December 2019. We determined laboratory-specific proportions per histologic grade and unadjusted odds ratios (ORs) for International Society of Urological Pathologists Grades 1 vs. 2–5 for 40 laboratories due to treatment implications for higher grades. Pathologist-specific proportions were determined for 21 laboratories that consented to this part of analysis. The synoptic reports of 21 laboratories were used for analysis of case-mix correction for PSA, age, year of diagnosis, number of biopsies and positive cores. Results: A total of 38,321 reports of 35,258 patients were included. Grade 1 ranged between 19.7% and 44.3% per laboratory (national mean = 34.1%). Out of 40 laboratories, 22 (55%) reported a significantly deviant OR, ranging from 0.48 (95% confidence interval (CI) 0.39–0.59) to 1.54 (CI 1.22–1.93). Case-mix correction was performed for 10,294 reports, altering the status of 3/21 (14%) laboratories, but increasing the observed variation (20.8% vs. 17.7%). Within 15/21 (71%) of laboratories, significant inter-pathologist variation existed. Conclusion: Substantial variation in prostate cancer grading was observed between and within Dutch pathology laboratories. Case-mix correction did not explain the variation. Better standardization of prostate cancer grading is warranted to optimize and harmonize treatment.

## 1. Introduction

Prostate cancer (PCa) is the most common cancer in European men, accounting for approximately one out of five newly diagnosed malignancies in men [1]. Patient numbers have tripled in thirty years, reaching approximately 13,600 newly diagnosed cases of prostate cancer in 2019 in The Netherlands [2]. Histologic grade is one of the best-established prognostic factors in PCa and is strongly associated with PCa-specific survival [3,4]. Diagnosis is mostly based on systematic ten to twelve core transrectal, ultrasound-guided (TRUS) prostate needle biopsies [5].

The universally recommended grading for PCa is the Gleason grading system [5]. The 2014 International Society of Urological Pathology (ISUP) conference adjusted Gleason grade groupings in order to better reflect the prognostic value of each Gleason grade class and to reduce observer variability [6]. This resulted in a more detailed description of architectural patterns for Gleason patterns 3–5 and a five-tier system (i.e., ISUP Grade 1 = 3 + 3, ISUP Grade 2 = 3 + 4, ISUP Grade 3 = 4 + 3, ISUP Grade 4 = sum score 8, and ISUP Grade 5 = sum score 9–10) [6].

Accuracy, consistency, and reproducibility in PCa grading by pathologists are essential for identifying patients who can benefit from active treatment or adjuvant oncological systemic therapy. Considerable interobserver variation has already been shown in smaller studies [7,8,9,10,11,12]. However, most did not reflect real-life grading in daily clinical practice. Previously, nationwide daily clinical practice studies showed considerable variation between Dutch pathology laboratories and individual pathologists within these laboratories for colorectal adenomas, colorectal adenocarcinomas, and ductal carcinoma in situ and invasive cancer of the breast (IBC) [13,14,15]. Additionally, specific interventions have successfully been performed in order to reduce interlaboratory grading variability using feedback reports and e-learning modules [16,17,18].

Therefore, we anticipated the possible presence of variation between pathology laboratories and between individual pathologists with regard to grading of PCa. To gain insight, awareness, and a baseline for future interventions in grading variation of PCa, we assessed the variation in histologic grading of over 35,000 patients with PCa between Dutch pathology laboratories and between individual pathologists within these laboratories using nationwide data from both synoptic and narrative pathology reports from daily pathology practice.

## 2. Materials and Methods

### 2.1. Data Source and Study Population

Data were extracted from the nationwide network and registry of histo- and cytopathology in The Netherlands (PALGA) database, which contains excerpts of all histology and cytology reports from Dutch pathology laboratories since 1991 [19]. Additionally, data regarding PSA value at diagnosis were extracted from The Netherlands Cancer Registry for the years 2017–2019 (IKNL) [20]. All extracted data from the PALGA database and IKNL were pseudonymized by a trusted third party (ZorgTTP, Houten, The Netherlands) and therefore did not contain direct identifiable data. All laboratories gave consent for storage and scientific use of their data in the PALGA database, which were anonymized. We obtained additional consent for evaluation of interpathologist variation within individual laboratories. The scientific and privacy committees of PALGA and IKNL approved of this study. All data were retrieved and handled in compliance with the General Data Protection Regulation Act (GDPR).

We retrieved all pathology reports of PCa needle biopsies between 1 January 2017 and 31 December 2019 in The Netherlands (*n* = 42,774). Since the majority of all PCa needle biopsies are still reported in an unstandardized, narrative report after the gradual implementation of synoptic reporting in 2016, we included both narrative (*n* = 29,377 (69%)) and synoptic reports (*n* = 13,397, (31%)). Only original prostate needle biopsy reports were included, thereby excluding 3245 re-evaluations, 52 transurethral resection reports, and 231 biopsies taken outside the prostate. As radiotherapy or antihormonal systemic treatment prior to needle biopsy can influence histologic grading, all pathology reports that mentioned patients whom had previously received these treatments were excluded (*n* = 105) [21,22,23]. Finally, reports without Gleason scores were excluded (*n* = 748). For analysis of interlaboratory variation, we excluded one laboratory, because it did not report PCa cases in 2019. For interpathologist variation within individual laboratories, we analyzed data for all pathologists who reported ≥20 PCa during the study period, concordant with previous intralaboratory studies [13,14,15,16].

### 2.2. Data Extraction

From the synoptically reported PCa cases, we extracted age at diagnosis, year of diagnosis, number of prostate biopsies taken and number of positive prostate biopsies, and Gleason grade. Missing values were excluded case-wise. From the narratively reported PCa cases, we extracted Gleason grades through regular expressions using the stringr R package [24]. An algorithm was created for each combination of Gleason grades (i.e., 3 + 3, 3 + 4, 3 + 5, 4 + 3, 4 + 4, 4 + 5, 5 + 3, 5 + 4, and 5 + 5). When reports described multiple Gleason grades, we extracted all and then chose the highest Gleason grade, as it was shown that the highest Gleason score on a given core correlates better with stage than the average or most frequent grade [23]. We validated this algorithm in a test set of randomly selected cases, where we compared the regular-expressions-generated Gleason grades to the manually extract the grades of 931 randomly selected cases, which revealed 99.4% agreement between the two approaches (unweighted kappa = 0.992). Other variables, as extracted from the synoptically reported PCa cases, were not extracted from the narrative reports due to high percentages of missing data in the test set of 931 cases.

### 2.3. Analysis of Histologic Grading

We analyzed the histologic grade according to the ISUP 2014 Grade Group classification [6]. The primary outcome measure of our study was the variation between pathology laboratories in ISUP Grade. For analysis, we dichotomized the outcome in ISUP Grades 1 versus 2–5, as patients with ISUP Grade 1 are considered for active surveillance, whereas international guidelines favor active treatment for higher grades [5]. The secondary outcome measures were the influence of case-mix variables on variation in a subset of the synoptic reports and the variation between pathologists within individual laboratories.

For analysis of case-mix variables, we used a subset of the synoptic reports that could be linked to the IKNL dataset (*n* = 11,733) as within the manually extracted dataset of 931 randomly selected cases, too many variables were missing (not at random) to be further used as case-mix variables. As PSA was only known for the primary diagnosis, but not for follow-up biopsies, we excluded follow-up biopsies for this part of the analysis (*n* = 977) and cases with no known PSA prior to diagnosis (*n* = 14). Laboratories reporting less than 100 synoptic reports for this period were excluded (*n* = 20). In total, 10,294 cases in 20 laboratories were analyzed for the influence of case-mix correction.

### 2.4. Statistical Analysis

Patient and tumor characteristics were summarized and differences between ISUP Grade 1 reports and ISUP Grades 2–5 reports were tested by means of a chi-square test for categorical variables and by Student’s *t*-test or Mann–Whitney-U test for continuous variables as appropriate.

**Figure 1:**
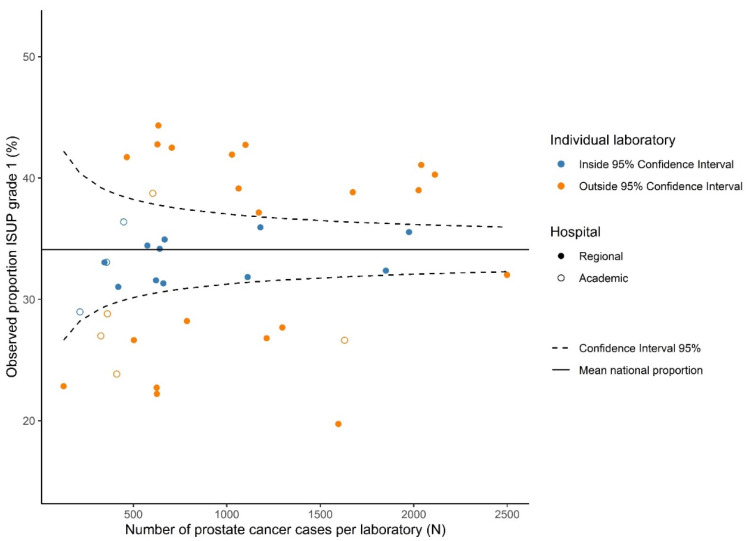
Funnel plot showing the observed proportion for ISUP Grade 1 prostate cancer grade per laboratory (dots) relative to the mean national proportion and its 95% confidence intervals.

We used the overall mean proportion for ISUP Grade 1 with corresponding 95% confidence intervals (CIs) as the national proportion. Absolute differences in proportions of histologic grade are presented in a funnel plot (Figure 1), in which the national mean proportion of ISUP Grade 1 is displayed with the corresponding 95%-CI. Subsequently, the proportion of ISUP Grade 1 per laboratory was plotted against the total number of PCa cases reviewed per laboratory.

**Figure 2:**
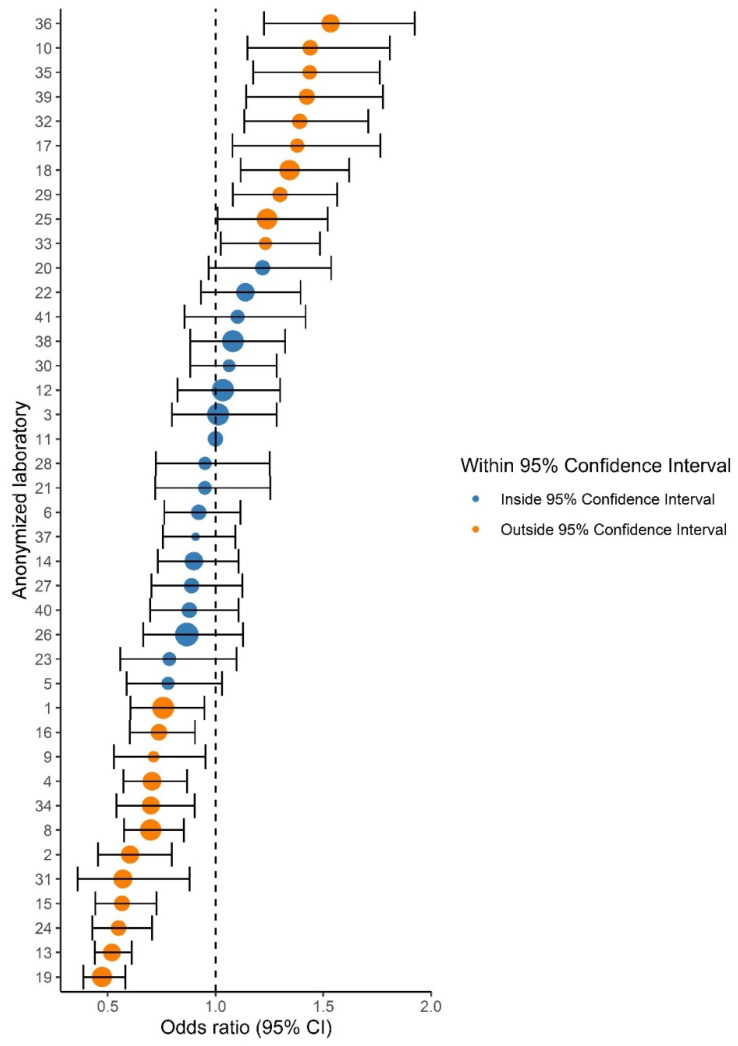
Forest plot showing the adjusted odds ratios (ORs) and 95% confidence intervals (CIs) of ISUP Grades 1 versus 2–5 prostate cancer grade in comparison to the reference laboratory (#11). Dot size indicates the total number of reported prostate cancers per laboratory. Orange dots indicate laboratories with a significantly deviant OR compared to the reference laboratory.

For comparing relative differences between laboratories, odds ratios (ORs) and 95% CIs were calculated by logistic regression and are presented in a forest plot (Figure 2). The reference category was the laboratory with a proportion of ISUP Grade 1 closest to the national mean.

A multivariable logistic regression was performed to analyze the effect of case-mix variables on laboratory-reported variation and to show the difference between adjusted and unadjusted proportions. Potential case-mix variables were selected a priori based on literature and experts’ opinions. These factors included PSA at diagnosis, age at diagnosis, year of diagnosis, total number of cores per biopsy, and total number of positive cores per biopsy [25,26,27,28].

For analysis of the interpathologist variation within individual laboratories, we compared the proportions of ISUP Grade 1 among pathologists by chi-square test.

All analyses and data manipulation were performed in R version 3.6.1 (R Foundation, Vienna, Austria) [29].

## 3. Results

In total, 38,321 reports of 35,258 unique patients were included. For 3063 patients, we included two or more pathology reports, as these concerned repeated prostate biopsies, as part of the follow-up regimen. All patients originated from a total of 40/41 Dutch pathology laboratories (as one laboratory did not grade PCa in 2019), grading 127–2499 (median 664) PCa lesions per laboratory. The characteristics of these patients are listed in Table 1. Mean age at diagnosis was 69.9 years. The proportion of synoptically reported PCa lesions per laboratory ranged from 0–89.9% (median 25.3%). The total proportion of synoptically reported PCa lesions was 33.8%. The number of laboratories that reported >75% as synoptic reports raised from 0 to 19 from 2017–2019, whereas the number of laboratories reporting <25% as synoptic reports lowered from 31 to 19 laboratories.

### 3.1. Interlaboratory Differences in ISUP Grading

Variation in ISUP grading existed throughout all grades, as 20–26/40 laboratories reported proportions outside the 95% CI for ISUP Grades 1–5. Laboratory-specific proportions ranged from 15.6–30.9% per grade (Table 2). The funnel plot for ISUP Grade 1 (vs. ISUP Grades 2–5) is shown in Figure 1. ISUP Grades 2–5 showed similar patterns (Figure S1). Proportions of ISUP Grade 1 per laboratory ranged from 19.7–44.3%. Twenty-six (65%) laboratories reported proportions outside the 95% CI based on the national mean of 34.1%. Laboratory 11 had the lowest deviation from the national mean proportion of ISUP Grade 1 (+0.07%) and was chosen as the reference laboratory. Logistic regression showed that 22 laboratories (55.0%) reported a significantly higher or lower proportion of ISUP Grade 1 cases than the reference laboratory (Figure 2). ORs of individual laboratories ranged from 0.47 (95% CI 0.39–0.58) to 1.53 (95% CI 1.22–1.92).

### 3.2. Case-Mix Correction

We analyzed the adjusted proportions and unadjusted proportions of ISUP Grade 1 for a subset of reports (*n* = 10,294). The patient and tumor characteristics used as case-mix variables are displayed in Table 3. Before case-mix correction, 11/21 laboratories (52.3%) had an unadjusted ISUP Grade 1 proportion outside the 95% CI. The range for unadjusted proportions was 23.7–41.4%. After case-mix correction, three laboratories shifted from outside to inside the 95% CI. All in all, 8/21 laboratories (38.1%) had an adjusted ISUP Grade 1 proportion outside the 95% CI after case-mix correction. The range of adjusted proportions was 22.7–43.5%. The median difference between the adjusted and unadjusted proportion of laboratories was 0.3% (Q1:Q3 = −1.2%:+1.8%, range = −6.7: +4.2). The laboratories that shifted to the 95% CI had a difference between adjusted and unadjusted proportions of 1.8%, 2.6%, and 4.2%, separately.

### 3.3. Intralaboratory Differences in Histologic Grading

**Figure 3:**
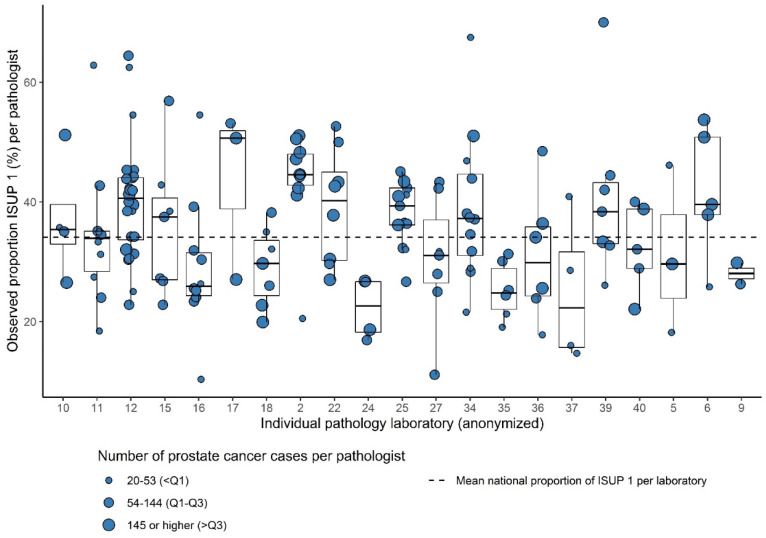
Box plots showing the observed proportion of ISUP Grades 1 versus 2–5 per pathologist (dots) within 21 laboratories relative to the mean national proportion for ISUP grade.

Of the 199 pathologists from the 21 laboratories, who consented to this part of analysis, 157 reported ≥20 tumors during the study period (78.8%). The total number of analyzed PCa cases for interpathologist variation was 18,264. The number of analyzed pathologists per laboratory ranged from 2 to 24 (median = 7). The number of PCa cases per pathologist ranged from 20 to 545 (median 99). Within 15/21 laboratories (71.4%), significant intralaboratory variation existed. Most variation was observed between the pathologists in laboratory 34 (range of proportion of ISUP Grade 1 21.6–67.5% per pathologist) (Figure 3).

## 4. Discussion

Using nationwide data, we analyzed the variation in the daily grading practice of PCa needle biopsies between and within Dutch pathology laboratories. We highlighted the substantial variation observed in ISUP Grade 1, as this is clinically relevant for the choice of active surveillance or active treatment, but similar substantial variation existed throughout all ISUP Grades. In our cohort of 38,321 cases, 13,067 cases (34.1%) were reported as an ISUP Grade 1 tumor. We performed both absolute and relative analyses on laboratory-specific data, comparing individual laboratories to the national mean proportion and a reference laboratory. Considerable grading variation was shown by the large range of proportions in ISUP Grade (proportions of ISUP Grade 1 ranged from 19.7–44.3%) and by the finding that more than half of the laboratories graded significantly deviated from the reference laboratory. Even though selection of the mean laboratory was arbitrary, as the reference laboratory does not necessarily diagnose PCa with greater accuracy, it was considered the best possible way to study interlaboratory variation.

The effect of case-mix correction on grading variation could only be analyzed for part of the synoptically reported PCa lesions (10,294 cases), since relevant case-mix variables were poorly reported in a randomly and manually extracted set, and not all cases could be linked to the IKNL database for PSA analysis. As a control, we compared ISUP Grade 1 proportions in the subset and the complete dataset and observed a similar distribution (*p* = 0.24). We therefore considered it representative of our complete dataset, allowing us to truly analyze nationwide data.

We tested the effect of case-mix correction by analyzing PSA at diagnosis, year of diagnosis, age, number of biopsies taken, and number of positive biopsies. All variables, except for year of diagnosis, were statistically significant. An individual laboratory close to the CI limits may shift toward the 95% CI of the national mean, since case-mix correction led to a shift of a few percentages in the adjusted proportions. However, case-mix correction within synoptically reported PCa did not decrease overall variation within our subset. The total range of proportions even increased after case-mix correction (20.8% vs. 17.7%). Therefore, we are confident that the univariate proportions are valid as reported.

A limitation of our case-mix correction is that we could not assess the role of all potential case-mix variables due to the retrospective nature of the data. For example, we could not assess the role of tumor volume, as it was either noted as tumor volume percentages or tumor length in millimeters, thereby leading to incomparable volume values. Other potential factors, such as lymphovascular invasion, prostate volume, or use of 5-alpha-reductase-inhibitors, were also unknown in our dataset and could potentially have influenced tumor grading [30,31,32,33]. However, given the limited influence of all other case-mix variables on the observed variation, we have no reason to assume that hypothetical differences in these variables would have had a major impact on the observed variation on a national level. In addition, on a national level, any potential influence of these and other case-mix factors on the overall observed variation is expected to be small as well, since it would only influence individual laboratories.

Another limitation is that it was largely unknown whether target biopsies or random biopsies were taken. Target biopsies or random biopsies can influence grading variation, as random biopsies can result in undergrading compared to target biopsies [26,34]. This is illustrated by the finding that concordance rates between biopsy and radical prostatectomy specimens improved after multiparametric magnetic resonance imaging-targeted biopsies [35,36]. It is possible that part of the variation was due to sampling variation. It is, however, unlikely that all variation can be attributed to the usage of target or random biopsies, as this study is the third analysis to find a substantial variation in national cancer grading [13,14,15].

In addition to interlaboratory variation, we observed a significant interpathologist variation within 15/21 of the analyzed laboratories (71.4%). This shows that widespread variation exists even within laboratories. Both pathologists reporting high volumes of PCa cases and pathologists reporting low volumes of PCa cases showed substantial variation.

Previous studies have already established interobserver variability in prostate cancer grading [7,8,10,12]. Allsbrook et al. found a moderate kappa of 0.435 for the Gleason grading system, and Ozkan et al. established considerable interobserver variability after the ISUP 2014 alterations (concordance of 51.7% and kappa = 0.39 for ISUP Grade) [7,10,12]. Santvoort et al. studied all revised pathology reports in The Netherlands from October 2015 until April 2016. They found that 25% of reports were up- or downgraded on revision, but the number of patients with re-evaluations was low (172 versus 5042 cases without re-evaluation, 3% and 97%, respectively) [8].

Our paper underlines that the known interobserver variability also translates to significant institutional variation in PCa grading. Clinicians should consider this when making treatment choices. Grade is especially determinant of local therapy for patients with a PSA <10, which was almost half of the PSA values known in our dataset [5]. It is therefore likely that grading variation has had a serious impact on treatment choice and thereby perhaps patient outcome. Van Santvoort et al. suggested that for one out of eight patients with localized PCa, grade re-evaluation might change treatment strategy [8]. No recommendations currently exist in the Dutch and EAU guidelines regarding grading re-evaluations.

Future research should focus on means of reducing grading variation in daily practice. This may be achieved by standardizing PCa grading in order to increase data and identify areas where grading variation occurs the most, so specific interventions can be applied. However, standardization will not be able to solve all grading variation. The modified Bloom–Richardson score for IBC is a (more) standardized way of tumor grading in IBC. Even so, Van Dooijeweert et al. found that significant variation between laboratories existed for IBC grading as well [13]. Therefore, we also suggest using feedback reports to enable pathologists to discuss and reflect on their grading practice. This can lead to regression to the mean as shown before [17]. In addition, training of pathologists through e-learning may reduce variation [17,37,38]. Another way to reduce interobserver variation is the adoption of artificial intelligence, which shows promising results [39,40,41,42].

## 5. Conclusions

In conclusion, this large nationwide cohort of PCa cases demonstrates considerable interlaboratory grading variation between and interpathologist variation within Dutch pathology laboratories. This likely affects treatment choice and prognosis. Better standardization of grading practice is needed for the optimal determination of prognosis and treatment choice.

## Figures and Tables

**Table 1:** Descriptive statistics of the full nationwide Dutch cohort of prostate cancer patients analyzed for grading variation.

	Total (*n* = 38,321)	ISUP Grade 1 (*n* = 13,067)	ISUP Grade 2–5 (*n* = 25,254)	*p*
Age (years) mean (SD)	69.9 (7.5)	67.9 (7.0)	70.9 (7.6)	*<0.001* ^a^
Year, *n* (%)				*<0.001* ^b^
2017	11,962 (31.2)	4434 (33.9)	7528 (29.8)	
2018	12,681 (33.1)	4243 (32.5)	8438 (33.4)	
2019	13,678 (35.7)	4390 (33.6)	9288 (36.8)	
Report type, *n* (%)				0.23 ^b^
Synoptic	12,954 (33.8)	4529 (34.7)	8425 (33.4)	
Narrative	25,367 (66.2)	8538 (65.3)	16,829 (66.7)	

ISUP = International Society of Urological Pathologists; *p* = statistically significant < 0.05; Q1–Q3 = interquartile range; ^a^ Student’s *t*-test; ^b^ χ^2^-test.

**Table 2:** Variation in ISUP Grade of patients in a nationwide Dutch cohort of prostate cancer patients in 40 laboratories.

	Mean Proportion (%)	Lowest Proportion per Laboratory (%)	Highest Proportion per Laboratory (%)	Total Range (%)	Number of Laboratories Outside 95% Confidence Interval *n*, (%)
ISUP Grade 1	33.5	19.7	44.3	24.6	26 (65.0)
ISUP Grade 2	23.4	10.2	36.1	25.9	21 (52.5)
ISUP Grade 3	13.7	7.1	22.7	15.6	20 (50.0)
ISUP Grade 4	12.9	4.8	26.4	21.6	21 (52.5)
ISUP Grade 5	15.9	6.1	37.0	30.9	25 (62.5)

ISUP = International Society of Urological Pathologists 2014 Grade.

**Table 3:** Descriptive statistics of the synoptically reported subgroup of patients in a nationwide Dutch cohort of prostate cancer patients analyzed for grading variation.

	Total (*n* = 10,294)	ISUP Grade 1 (*n* = 3228)	ISUP Grade 2–5 (*n* = 7066)	*p*
Age (years), mean (SD)	70.2 (7.6)	68.1 (7.1)	71.2 (7.6)	<0.001 ^a^
Number of cores, mean (SD)	9.6 (3.1)	10.1 (2.9)	9.9 (3.1)	<0.001 ^a^
Number of positive cores, median; (Q1–Q3)	4 (2–7)	2 (1–4)	5 (4–8)	<0.001 ^b^
Prostate-specific antigen, median (Q1–Q3)	10.8 (6.9–25.0)	7.7 (5.8–11.0)	14.4 (8.0–45.1)	<0.001 ^b^
Year of diagnosis, *n* (%)				0.01 ^c^
2017	1715 (16.7)	590 (18.3)	1125 (15.9)	
2018	3763 (36.6)	1160 (35.9)	2603 (36.8)	
2019	4816 (46.8)	1478 (45.8)	3338 (47.2)	

ISUP = International Society of Urological Pathologists; *p* = statistically significant < 0.05; Q1–Q3 = interquartile range; ^a^ Student’s I-test between ISUP Grades 1 and ISUP 2–5; ^b^ Mann–Whitney U-test between ISUP Grades 1 and ISUP 2–5; ^c^ χ^2^-test.

## Data Availability

Restrictions apply to the availability of these data. Data were obtained from PALGA and NCR and are only available from the authors with the permission of these institutions.

## Supplementary Materials

The following are available online at https://www.mdpi.com/article/10.3390/cancers13215378/s1, Figure S1: Funnel plot showing the observed proportion for ISUP Grade 2 prostate cancer grade per laboratory (dots) relative to the mean national proportion and its 95% confidence intervals. Figure S2: Funnel plot showing the observed proportion for ISUP Grade 3 prostate cancer grade per laboratory (dots) relative to the mean national proportion and its 95% confidence intervals. Figure S3. Funnel plot showing the observed proportion for ISUP Grade 4 prostate cancer grade per laboratory (dots) relative to the mean national proportion and its 95% confidence intervals. Figure S4: Funnel plot showing the observed proportion for ISUP Grade 5 prostate cancer grade per laboratory (dots) relative to the mean national proportion and its 95% confidence intervals.
